# A Review on Forest Fire Detection Techniques: Past, Present, and Sustainable Future

**DOI:** 10.3390/s26051609

**Published:** 2026-03-04

**Authors:** Alimul Haque Khan, Ali Newaz Bahar, Khan Wahid

**Affiliations:** Electrical and Computer Engineering, University of Saskatchewan, Saskatoon, SK S7N 5B5, Canada; ali.bahar@usask.ca (A.N.B.); khan.wahid@usask.ca (K.W.)

**Keywords:** detection techniques, early detection systems, ecological challenges, forest fires, internet of things, remote sensing technologies, sensor networks, unmanned aerial vehicle

## Abstract

Forest fires are a major concern due to their significant impact on the environment, economy, and wildlife habitats. Efficient early detection systems can significantly mitigate their devastating effects. This paper provides a comprehensive review of forest fire detection (FFD) techniques and traces their evolution from basic lookout-based methods to sophisticated remote sensing technologies, including recent Internet of Things (IoT)- and Unmanned Aerial Vehicle (UAV)-based sensor network systems. Historical methods, characterized primarily by human surveillance and basic electronic sensors, laid the foundation for modern techniques. Recently, there has been a noticeable shift toward ground-based sensors, automated camera systems, aerial surveillance using drones and aircraft, and satellite imaging. Moreover, the rise of Artificial Intelligence (AI), Machine Learning (ML), and the IoT introduces a new era of advanced detection capabilities. These detection systems are being actively deployed in wildfire-prone regions, where early alerts have proven critical in minimizing damage and aiding rapid response. All FFD techniques follow a common path of data collection, pre-processing, data compression, transmission, and post-processing. Providing sufficient power to complete these tasks is also an important area of research. Recent research focuses on image compression techniques, data transmission, the application of ML and AI at edge nodes and servers, and the minimization of energy consumption, among other emerging directions. However, to build a sustainable FFD model, proper sensor deployment is essential. Sensors can be either fixed at specific geographic locations or attached to UAVs. In some cases, a combination of fixed and UAV-mounted sensors may be used. Careful planning of sensor deployment is essential for the success of the model. Moreover, ensuring adequate energy supply for both ground-based and UAV-based sensors is important. Replacing sensor batteries or recharging UAVs in remote areas is highly challenging, particularly in the absence of an operator. Hence, future FFD systems must prioritize not only detection accuracy but also long-term energy autonomy and strategic sensor placement. Integrating renewable energy sources, optimizing data processing, and ensuring minimal human intervention will be key to developing truly sustainable and scalable solutions. This review aims to guide researchers and developers in designing next-generation FFD systems aligned with practical field demands and environmental resilience.

## 1. Introduction

Forest fires are an increasingly serious environmental concern worldwide. In 2024, some regions experienced extremely intense fire seasons, although total global area burned decreased slightly. According to the Copernicus Atmosphere Monitoring Service (CAMS), wildfire emissions in 2024 reached approximately 1940 megatonnes of carbon, driven primarily by record-breaking activity in the Americas [1]. In South America, exceptional drought conditions led to the most intense fire season in more than two decades. Nations such as Bolivia and Venezuela recorded their highest annual emissions in the CAMS dataset, with significant burning in the Amazon and Pantanal wetlands [1]. In contrast, fire activity in Southeast Asia remained below average, continuing a downward trend observed over the past two decades [1]. Africa, which accounts for the largest global burned area, has experienced a gradual decline in savanna fires due to landscape fragmentation and changes in land use. However, it remains a major contributor to global pyrogenic emissions [1,2].

These global trends illustrate the diverse ways wildfires impact forest ecosystems. Frequent and intense fires destroy habitats and reduce biodiversity, making it difficult for specialist species to survive [3]. Soil properties are also significantly altered; while low-intensity fires can enhance fertility through ash deposition, high-intensity fires severely damage soil structure and destroy essential soil organisms [4,5].

The increasing frequency, scale, and complexity of wildfire events have intensified the need for effective early detection and monitoring systems. Timely identification of fire outbreak and accurate situational awareness are critical for minimizing environmental damage, economic losses, and threats to human life [6]. Traditional fire monitoring approaches based on manual observation or basic remote sensing are no longer sufficient to address the spatial extent and dynamic behavior of modern wildfires [7]. Consequently, significant research efforts have focused on developing automated forest fire detection systems that integrate sensing technologies, communication networks, and intelligent data processing. These advances include ground-based IoT sensing systems [7], deep learning and computer vision methods for fire and smoke detection [8], and AI-enabled UAV platforms for enhanced monitoring [9], thereby enabling rapid, reliable, and scalable wildfire monitoring.

### 1.1. Methodology

This review follows a structured and transparent process to ensure reproducibility. To preserve generality, the number of records at each stage is reported as approximate values rather than exact counts. Accordingly, this work follows a structured narrative review approach supported by PRISMA-inspired screening, rather than a fully systematic review. The literature identification, screening, and synthesis steps are summarized below.

Relevant literature is retrieved from major academic databases, including IEEE Xplore, ScienceDirect, Web of Science, Scopus, and Google Scholar. These databases are selected for their broad coverage of engineering and applied sciences. Searches employed combinations of keywords such as “forest fire detection”, “wildfire detection”, “early fire detection”, “UAV”, “IoT”, “edge node”, “wireless sensor networks”, “autonomous sensing”, and “LoRa”, using Boolean operators (AND/OR) for refinement. Publications from 2000 to 2025 are primarily considered, with earlier studies included selectively to provide historical context.

This review primarily includes peer-reviewed journal articles, conference papers, and technical reports related to forest fire detection and monitoring. In addition, selected non-academic sources such as official websites, technical blogs, and online reports from credible organizations and companies (e.g., NASA, NOAA, DJI) are included when they provided essential technical details, system descriptions, or contextual information that is not available in formal publications. Sources that are irrelevant, lack credibility, or fall outside the scope of this review are excluded.

The literature screening is conducted in three sequential stages: (i) preliminary evaluation of titles and abstracts to exclude studies outside the scope of forest fire detection, (ii) detailed review of the full texts of the remaining publications, and (iii) final selection of studies demonstrating sufficient technical rigor and relevance to detection systems, methodologies, and applications.

**Figure 1:**
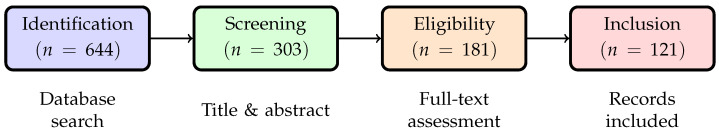
Overview of the literature review screening process and records kept at each stage.

Key quantitative parameters related to forest fire detection performance and system characteristics are systematically compared against multiple independent studies. This comparative verification enhances consistency and credibility. Consequently, summarized data reflect the range of values commonly reported in the literature. This review systematically progresses from broad forest fire detection challenges and opportunities, including historical perspectives, to advanced sensor, UAV, IoT, and AI-based detection frameworks, following a PRISMA-style screening and selection process (Figure 1).

### 1.2. Causes of Forest Fire

Forest fire occurrence is driven by a range of natural and human-induced factors, whose relative contributions have been documented through empirical studies and regional observations. Negligent use of fire accounts for a substantial proportion of fire incidents, particularly in regions with traditional agricultural practices such as crop residue burning and pasture renewal. This pattern of fire occurrence has been frequently reported in northern Portugal and across southern Europe [10,11].

Accidental ignitions are commonly linked to agricultural machinery, including harvesting equipment, as well as other unintentional human activities that increase fire risk [10]. Deliberate actions, including arson and intentional fire-setting motivated by economic or other gains, represent a major cause of forest fires. Expert assessments indicate a high prevalence, with estimates suggesting that nearly 40% of identified fires in Portugal fall into this category [10,11]. Natural causes, such as lightning strikes, occur less frequently than human-induced ignitions but remain a recognized source of fire outbreaks [12]. In addition, a considerable proportion of forest fires are classified as having unknown causes, highlighting persistent limitations in investigation, reporting, and data collection practices [10]. Environmental conditions such as drought and strong winds further influence fire ignition potential, spread, and intensity.

Wildfire ignition causes can be modeled as a function of multiple natural and anthropogenic factors, as expressed in Equation (1). Natural causes (*N*) are predominantly associated with lightning-induced ignitions and constitute a major source of wildfires in remote regions [13]. Anthropogenic causes include human negligence (*H*), such as unattended campfires or discarded cigarettes, which have been shown to significantly expand the spatial and temporal fire niche compared with natural ignitions [14]. Debris and biomass burning (*D*), including agricultural residue burning and land management practices, represent a major class of human-related fire causes at both regional and global scales [15]. Intentional ignition or arson (*A*) are commonly treated as a distinct anthropogenic category in national and regional fire databases [16]. Infrastructure-related causes (*I*), particularly failures of electrical power transmission systems, have been identified as critical ignition mechanisms responsible for several catastrophic wildfire events [17]. Finally, a non-negligible fraction of wildfire events remains classified as unknown or unclassified (*U*) due to insufficient post-fire evidence or reporting limitations, as documented in national and international fire statistics.

(1) Causes=f(N,H,D,A,I,U)

where:

*N* = natural causes (e.g., lightning, volcanic activity)

*H* = human negligence (e.g., campfires, discarded cigarettes)

*D* = debris or biomass burning (e.g., agricultural waste)

*A* = arson (intentional fire ignition)

*I* = infrastructure-related causes (e.g., power line sparks)

*U* = unknown or unclassified causes

### 1.3. Phases of Forest Fire

**Figure 2:**
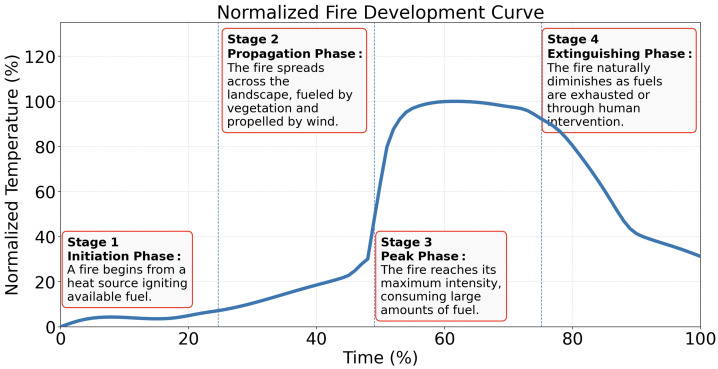
Normalized fire development curve showing the four stages of fire evolution.

While natural events such as lightning can initiate forest fires, human activities remain the primary drivers of their increasing frequency and severity. This rise in anthropogenic ignitions raises serious concerns about ecological imbalance and the acceleration of climate change effects [18]. Regardless of the ignition source, forest fires tend to follow a predictable developmental pattern. Understanding this progression is critical for effective fire modeling, risk assessment, and response planning. Although the causes of forest fires vary by region [19,20,21], their development often follows a common trajectory. The wildfire lifecycle can generally be divided into four core phases—ignition, propagation, peak intensity, and extinguishment—as illustrated in Figure 2 [22,23]. These stages broadly correspond to the more granular seven-phase model proposed by Viegas [24], which includes preheating, ignition, flaming combustion, transition, full development, decay, and cooling.

No single temperature–time curve can adequately represent all fire scenarios, as fire development is influenced by factors such as fuel load, ventilation conditions, compartment geometry, and suppression actions. Nevertheless, experimental studies and fire engineering models consistently show that most fires exhibit broadly similar temporal behavior, characterized by a growth phase, a fully developed phase, and a decay phase, largely independent of the specific fuel type involved. This common structure has been observed across compartment fire experiments and standardized fire tests, providing the basis for a widely used temperature–time relationships in fire safety engineering [25]. As a result, generalized fire curves are frequently employed for analysis and design purposes, while recognizing their inherent limitations in capturing the full complexity of real fire behavior. A normalized fire development curve is shown in Figure 2 [26]. A fire is easiest to control during the initiation phase, as it remains small and localized, making it more manageable to extinguish before it spreads.

### 1.4. The Complete Cycle of a Forest Fire

Forest fires progress through a well-defined life cycle that begins with a pre-ignition phase, during which combustible fuel accumulates on the forest floor and dries under favorable environmental conditions, leading to fuel preheating and a reduced ignition energy threshold [27]. Ignition occurs when a heat source initiates combustion in dry fuel, triggering flame propagation. This is followed by the combustion stage, during which flaming combustion releases heat, light, and smoke as available fuel is consumed, while smoldering combustion may persist under low-oxygen conditions, producing smoke and glowing embers. A fire reaches extinction when fuel is exhausted or suppression efforts succeed, allowing the affected area to cool and reducing the likelihood of re-ignition. In the post-fire stage, ecological recovery begins, with regeneration processes varying depending on fire severity, ecosystem resilience, and prevailing environmental conditions [28].

Forest fires can be classified into several categories based on their cause, cause, materials burned, size, and environment. By cause, fires may originate from natural phenomena such as lightning strikes or volcanic eruptions, or they may result from human activities, including arson and unattended campfires [29]. Another way to categorize fires is by the type of material that burns. Ground fires occur in the humus or organic materials beneath the surface, while surface fires consume loose debris on the forest floor, such as dead leaves and grass [28]. Crown fires, on the other hand, spread through the canopy and burn the tops of trees [30].

Classification by size typically ranges from Class A, representing the smallest fires, to Class E, which includes the largest fires. Detailed definitions may vary by region and are often outlined by firefighting agencies. Fires can also be distinguished by the environment in which they occur: wildfires generally affect rural and wilderness areas, whereas urban fires occur near or within cities and suburban regions [31]. Finally, fire behavior provides another basis for classification. Smoldering fires are low-intensity and slow-moving [32], creeping fires advance gradually across the forest floor, and running fires spread rapidly, driven by strong winds and dry conditions [33].

### 1.5. Global Trends in Forest Fire Frequency and Impact

The rising frequency and intensity of forest fires present serious environmental, social, and economic risks [34]. In Balgazyn stands, the mean fire intervals are 10.4 years, compared to 22.4 years at the landscape scale [35]. Shortened fire intervals in southern Siberia are causing major forest transformations [36], while boreal forests in northern Alberta show strong spatial and temporal variability influenced by local geography [37]. Long-term data from Kootenay National Park further link changes in fire frequency to climate variability and human activity [38]. The 2025 season showed early onset and extreme severity, with burned areas far exceeding the decadal average [39,40]. In the United States, although annual ignitions have slightly declined since 2000, an average of 7 million acres burn annually, with lightning-ignited fires responsible for over half of the total burned area [41]. As wildfires increasingly encroach on developed areas, risk management must prioritize the Wildland–Urban Interface (WUI). Structural losses are largely driven by ember exposure and building-to-building ignition. Localized measures, such as fire-resistant construction and land-use planning, are essential to reduce catastrophic losses during high-intensity wildfires [42]. The growing complexity of wildfire management has driven the development of advanced Decision Support Systems (DSS) for tactical response and long-term planning under uncertainty [43]. Recent advances combine UAVs with deep learning to enable real-time smoke and fire detection, providing flexible, high-resolution monitoring in remote and complex environments [44].

## 2. Historical Practices in Forest Fire Detection

Forest fires have been a persistent concern throughout human history, imposing significant ecological and economic challenges worldwide. Efficient early detection, and in some cases prediction, can more effectively mitigate their devastating impacts. In ancient times, humans became aware of wildfires only when the fires were large enough to produce visible smoke and had already burned a large area. Watchtowers are still used [45,46] to monitor forest fires. The use of bi-objective optimization models to strategically position watchtowers marks a significant advancement in forest fire monitoring systems [47]. Spatial optimization of watchtower locations has proven vital for early fire detection in high-risk regions such as Thasos Island, enabling up to 70% visibility with minimal overlap [48].

In the early days of forest fire detection, elevated lookout towers were the primary line of defense. These towers relied heavily on human vigilance, with observers scanning the horizon for signs of smoke or fire. While this method provided a panoramic view, it was heavily constrained by human limitations, especially in extensive and remote forest regions. There are 1781 lookouts registered in the United States [49]. In Alberta, Canada, there are 127 lookout towers [50]. Although these towers enabled faster detection, they offer limited technological capability for fire suppression or disaster management. Recent technological advancements have improved early wildfire detection, enabling authorities to respond more rapidly. Towers that remain in operation still require continuous human monitoring. However, operators in many of them are now being replaced by multiple cameras and camera views are observed remotely to detect forest fires. The limitations of relying solely on human surveillance are particularly evident in vast and remote forested areas. Traditionally, fire lookout towers enabled wide-area monitoring from elevated positions but required continuous human presence and were constrained by visibility and weather conditions [51]. In recent years, many of these towers have been replaced by fixed surveillance cameras [52], which serve as modern counterparts to traditional systems. However, fixed cameras remain limited by their static coverage and infrastructure requirements. Mounting cameras on unmanned aerial vehicles (UAVs) addresses these limitations by offering mobility, rapid deployment, and real-time data acquisition [53]. Compared with satellites and stationary systems, UAV-based platforms provide a more flexible and cost-effective solution for large-scale forest fire detection and monitoring [54].

**Figure 3:**
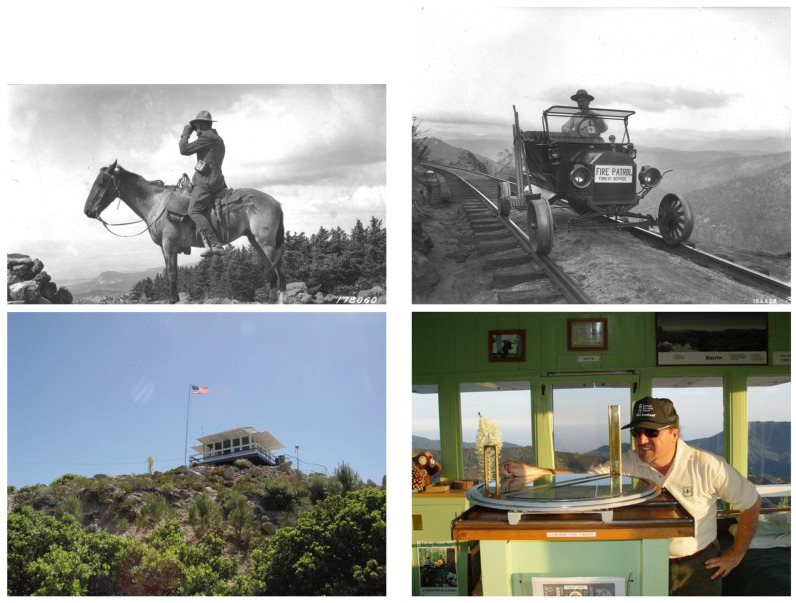
Historical human-based forest fire detection methods, including ground patrols and lookout towers, used before the adoption of automated monitoring systems [55,56].

The age-old practice of wildfire detection relied on elevated structures that provided a panoramic view of surrounding forested areas. These methods depended entirely on human vigilance and manual communication, often involving patrols on horseback or by rail. Early lookout towers enabled surveillance over large regions from elevated positions. However, such approaches were limited by human fatigue, delayed reporting, and restricted coverage, particularly in vast and remote forests. Examples of these historical human-based detection methods are shown in Figure 3. The evolution of fire detection systems has undergone notable transitions from mechanical to electronic solutions. In the early stages, telegraph-based alarms and bimetallic thermal sensors laid the groundwork for automated fire alerts.

The 20th century saw the introduction of smoke detectors and heat sensors that enabled real-time, localized fire detection [6]. These technologies marked a shift from manual observation to sensor-based automation, offering early advantages such as faster response times and reduced human workload. Despite these improvements, early systems were limited in scope. Their stationary nature reduced overall area coverage, and their performance could be impaired by environmental conditions, resulting in false alarms or delayed detection [57]. These shortcomings underscored the need for mobile, adaptable surveillance systems.

## 3. Modern Techniques for Forest Fire Detection

The field of forest fire detection has experienced a major transformation with the introduction of advanced tools and methodologies. Emphasis on precision, speed, and comprehensive coverage has led to the development of modern detection techniques. Satellite imaging has emerged as a powerful tool, providing real-time or near-real-time observations of large-scale landscapes [58]. Enhanced spatial resolution and spectral capabilities of these satellites provide unprecedented levels of detail [59]. Ground-based sensors complement this aerial perspective and demonstrate improved capabilities in detecting changes in temperature, infrared signatures, and smoke presence [60]. These sensors, often networked together, form integrated monitoring systems that continuously observe forest environments [61]. Automated camera systems further strengthened this network by continuously monitoring designated areas and, through AI integration, identifying potential threats in real time [6]. Meanwhile, drones and aircraft have become increasingly prominent, offering flexible, wide-area monitoring while transmitting real-time data to support rapid intervention [54]. The different sensor types and real-world system architectures are summarized in this section.

### 3.1. Ground-Based Sensors

**Figure 4:**
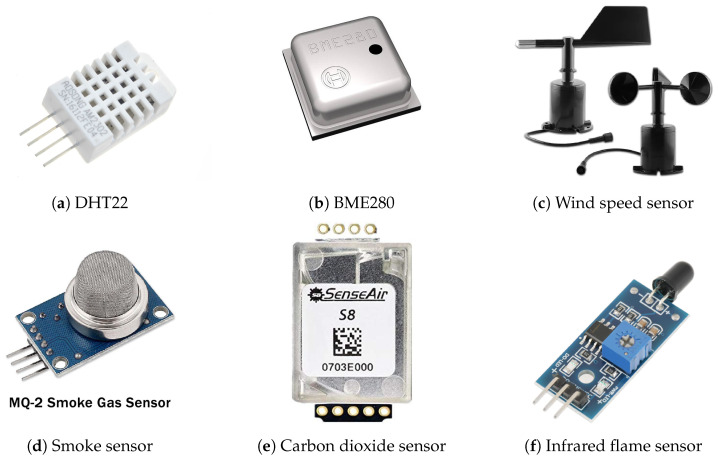
Commonly used sensors for forest fire detection (FFD) systems.

Ground-based sensors play a crucial role in modern forest fire detection by providing localized, accurate, and continuous monitoring of environmental conditions. These sensors are strategically placed in high-risk areas to detect critical indicators of fire, such as temperature spikes, smoke particles, and infrared radiation [7]. Sensors can measure several types of environmental parameters. Temperature sensors monitor thermal fluctuations, and an abrupt rise may indicate a potential fire. A system equipped with advanced algorithms can differentiate between false alarms and genuine fire risks, enabling accurate and timely responses [62]. Temperature sensors often integrate humidity measurements (Figure 4a) and, in some cases, barometric pressure sensing capabilities (Figure 4b). These sensors measure moisture content in the air, which is a critical factor in assessing fire risk. Low humidity levels can significantly increase the likelihood of fire ignition and spread [63,64]. An abrupt drop in humidity may be an early indication of a forest fire. However, both temperature and humidity should be considered when assessing fire conditions. To improve accuracy, more datasets are required. Wind is a critical factor influencing the rapid expansion of wildfires. It can be measured using a wind sensor (Figure 4c) that provides both wind speed and direction. Although wind speed alone is not sufficient to detect fires, integrating wind sensors with other sensing modalities—such as temperature, humidity, and smoke—can significantly enhance fire detection accuracy and fire spread modeling [65].

Smoke sensors (Figure 4d) are designed to detect particles and gases typically emitted during combustion, such as carbon monoxide (CO) and particulate matter (PM). They are critical components in early detection systems, either as standalone units or integrated into larger surveillance networks [66,67]. Carbon dioxide sensors (Figure 4e) are available to detect carbon dioxide and, in some cases, carbon monoxide or other combustion-related gases. Infrared sensors detect thermal radiation (Figure 4f), which serves as the primary indicator of fire. These sensors can identify heat signatures even in low-visibility conditions, such as dense fog or at night, making them particularly effective for early-stage fire detection [68,69]. A comparative summary of representative ground-based sensors and their primary characteristics used in forest fire detection systems is presented in Table 1.

Given the complementary capabilities and inherent limitations of individual sensors, reliable wildfire monitoring cannot rely on a single sensing modality, highlighting the importance of sensor fusion. Sensor fusion refers to the systematic integration of data from multiple heterogeneous sensors to achieve a more accurate, reliable, and comprehensive interpretation of an observed event than can be obtained from any single sensor [70]. In the context of wildfire monitoring, sensor fusion integrates complementary data from thermal, optical, multispectral, gas, meteorological, and other environmental sensors. This approach enhances detection robustness and reduces false alarms under diverse operating conditions [71]. Fusion frameworks combine thermal, optical, multispectral, and meteorological data to enhance situational awareness. This integration improves reliability across varying illumination, weather, and terrain conditions. Data-level, feature-level, and decision-level fusion strategies are commonly employed to merge sensor outputs, allowing early fire indicators to be identified even in the presence of noise, partial occlusion, or sensor uncertainty [72]. As a result, sensor fusion plays a critical role in modern wildfire detection systems by supporting timely event recognition, adaptive risk assessment, and reliable alert generation over large and heterogeneous forest environments.

### 3.2. Image Sensors

**Figure 5:**
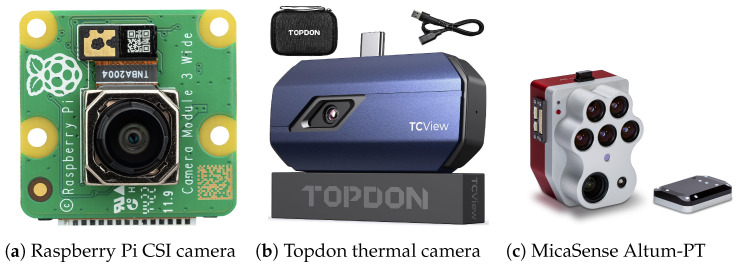
Examples of commonly used imaging sensors for wildfire monitoring, including (**a**) an optical camera, (**b**) a thermal camera, and (**c**) a multispectral camera.

Imaging-based sensing technologies are widely used in wildfire monitoring due to their ability to capture spatial, thermal, and spectral characteristics of fire-related events. Optical cameras are among the most common visual sensing technologies for forest fire detection. They capture scene information in the visible spectrum, similar to human vision, enabling the observation of smoke, flames, and changes in illumination. Optical camera-based systems are particularly effective for early smoke detection during the initial stages of a fire, when thermal signatures may still be weak. Image processing and computer vision techniques are commonly applied to extract motion, color, and texture features associated with smoke and flame behavior [73]. Standard devices such as mirrorless and DSLR models are typical examples of optical cameras. In addition, compact cameras can be directly integrated with microcontrollers or single-board computers to create dedicated fire detection systems. A Raspberry Pi camera, shown in Figure 5a, is an example of optical camera. Due to their low cost and ease of deployment, optical cameras are suitable for continuous monitoring of forested areas from watchtowers, fixed installations, and unmanned aerial platforms. However, their performance can be affected by lighting variations, weather conditions, and visual obstructions. Therefore, robust algorithmic support is required to ensure reliable fire detection [74].

A thermal camera can be considered a multi-array infrared sensor, whereas a conventional infrared sensor can be treated as a 1 × 1 thermal array. Widely used research-grade thermal sensor arrays provide low spatial resolution, typically 32 × 24 or 64 × 48 pixels. Devices such as the Melexis MLX90640 and MLX90641 are favored for their low power consumption and direct access to raw temperature data, supporting algorithm development and embedded processing. In contrast, commercial thermal cameras from manufacturers such as TOPDON (shown in Figure 5b), FLIR, Seek Thermal, and HIKMICRO provide higher spatial resolution and advanced image enhancement capabilities, but are often coupled with proprietary hardware and software. These thermal cameras capture temperature distributions over defined areas, where abnormal thermal patterns are analyzed to identify hotspots indicative of potential fire outbreaks. Equipped with advanced algorithms, such systems can distinguish between false alarms and genuine fire risks, enabling accurate and timely responses [67,75].

Multispectral cameras capture multiple wavelength bands, including visible, near-infrared (NIR), and short-wave infrared (SWIR), enabling detailed analysis of surface and vegetation characteristics. Figure 5c shows an example of a MicaSense Altum-PT multispectral camera. By monitoring spectral signatures related to chlorophyll content, moisture stress, and canopy structure, these sensors can detect early changes in vegetation health that may indicate increased fire susceptibility. Indices derived from multispectral data, such as vegetation vigor and moisture-related metrics, enable the identification of drought-stressed or degraded areas that are more prone to ignition and rapid fire spread. Owing to their wide spatial coverage and temporal monitoring capabilities, multispectral sensors support proactive fire risk assessment and long-term forest condition analysis. They thereby complement thermal and point-based sensing systems within comprehensive wildfire prevention and monitoring strategies [76]. Table 2 summarizes the key characteristics and comparative features of the main imaging sensor modalities used in forest fire detection systems.

### 3.3. Automated Camera Systems

**Figure 6:**
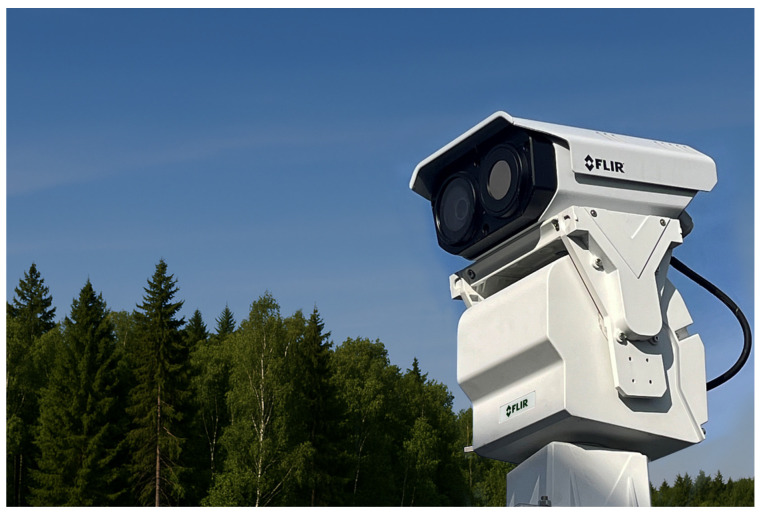
FLIR FH-Series thermal–visible PTZ camera for early fire detection and monitoring.

Automated camera systems have significantly improved forest fire detection by enabling continuous surveillance and real-time analysis over large forested areas. Deployed at elevated towers or natural vantage points, these systems provide wide-area coverage and continuous monitoring without constant human involvement. Modern deployments commonly use optical, multispectral, and infrared (IR) cameras, often enhanced with artificial intelligence to automatically detect smoke, flames, or abnormal thermal patterns, reducing false alarms and supporting early response. In advanced configurations, thermal cameras perform wide-area scanning to identify potential hotspots, while automated pan–tilt–zoom (PTZ) visible-light cameras are directed to the target location for visual confirmation and detailed inspection, as illustrated in Figure 6. Such systems typically integrate imaging sensors, edge-level processing, PTZ control, and communication modules to generate real-time alerts. A representative example is the FLIR TrafiBot Dual AI camera, which combines a high-sensitivity thermal core with embedded early fire detection capabilities, enabling reliable detection in complex environments [77]. Overall, automated camera-based systems offer scalable, continuous monitoring over large areas, although their performance remains influenced by field-of-view limitations and infrastructure availability.

### 3.4. Aerial Surveillance Through Drones and Aircraft

Aerial surveillance platforms, including unmanned aerial vehicles (UAVs) and manned aircraft, have become essential components of modern forest fire detection systems by enabling mobile, wide-area monitoring with high temporal flexibility [54,78]. Unlike fixed ground-based sensors or satellite platforms, aerial systems can be rapidly deployed over diverse and often inaccessible terrain, providing real-time situational awareness that supports early fire detection and emergency response [79]. Operating above forest canopies, these platforms facilitate early identification of thermal anomalies, smoke plumes, and fire fronts, particularly in regions where stationary sensing systems suffer from limited coverage or line-of-sight constraints [80].

Drones and manned aircraft offer complementary capabilities in aerial wildfire monitoring. UAVs enable low-altitude, high-resolution surveillance and can be equipped with optical, thermal, multispectral, and environmental sensors for localized inspection of high-risk zones at a comparatively low operational cost [78,81]. In contrast, manned aircraft provide extended flight endurance and broad-area coverage, making them suitable for regional-scale monitoring and tracking fire propagation during large wildfire events [54,79]. The integration of aerial platforms with ground-based sensing infrastructure and fixed camera systems enables a multilayered detection architecture. As a result, coverage gaps are reduced, and overall detection reliability is improved.

Recent advancements in wildfire monitoring have increasingly leveraged UAVs in combination with machine learning, wireless sensor networks, and Internet of Things (IoT) technologies to improve detection accuracy, reduce false alarms, and enable near-real-time decision-making [82]. UAVs equipped with advanced imaging sensors and onboard artificial intelligence have demonstrated faster fire detection than certain satellite-based systems, particularly for small or early-stage fires. Moreover, UAV deployment costs are significantly lower than those associated with satellite missions or the installation and maintenance of extensive lookout tower infrastructure [83].

Several studies have explored cooperative and intelligent UAV-based wildfire monitoring frameworks. Distributed control systems have been proposed to coordinate multiple UAVs for dynamic tracking of wildfire fronts, enabling flexible and safe operation in hazardous environments [84]. UAV-assisted IoT networks have demonstrated strong potential to provide faster and more reliable wildfire detection than traditional satellite-based approaches, particularly under optimized deployment strategies [85]. To address data scarcity challenges, drone-collected RGB and thermal image datasets have been introduced to support early fire detection and deep learning-based segmentation in remote forest regions [86]. Additionally, UAV-based systems integrating air quality sensors and LiDAR have been developed for early wildfire detection, incorporating energy-optimized patrolling strategies and plume dispersion modeling [87].

### 3.5. Satellite Imaging

Satellite imaging plays a complementary role in forest fire detection by providing large-scale and near-real-time observations of extensive forested regions. Modern Earth observation satellites equipped with optical and infrared sensors are capable of detecting active fires, thermal anomalies, and smoke plumes, thereby enabling early situational awareness over wide geographic areas [88]. Advances in spatial and spectral resolution have significantly improved monitoring of fire dynamics and burned areas, particularly through operational satellite products such as MODIS and VIIRS [89]. Despite these advantages, satellite-based fire detection faces inherent limitations that restrict its effectiveness for real-time operational response. Temporal constraints arising from satellite revisit intervals, data processing delays, and transmission latency can cause delays in fire detection, particularly for rapidly evolving events [90].

**Figure 7:**
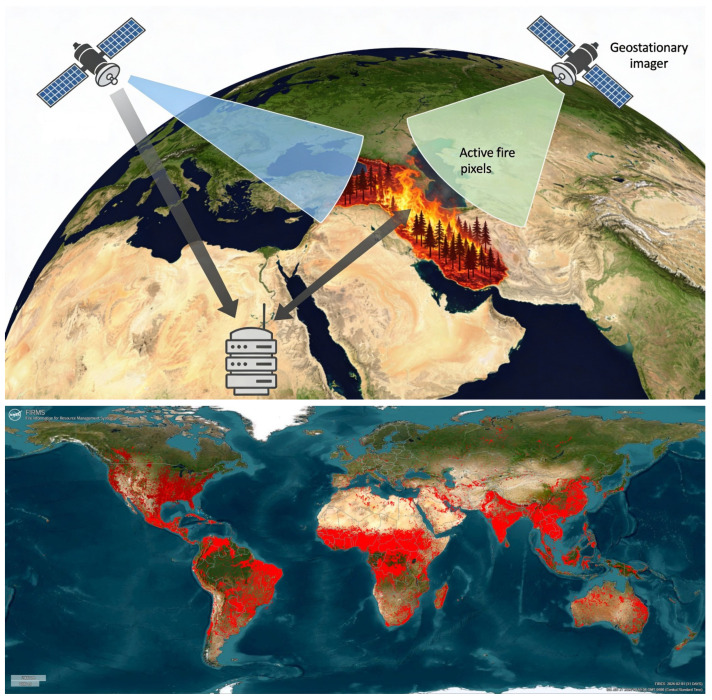
The principle of thermal anomaly detection using satellite images (**top**) and dashboard example of NASA’s FIRMS (**bottom**).

Additionally, cloud cover, dense smoke, and the coarse spatial resolution of some sensors may hinder the detection of small or low-intensity fires. For example, NASA’s Fire Information for Resource Management System (FIRMS) provides near-real-time global fire observations with typical latencies of up to several hours, which may be insufficient for immediate tactical decision-making in emergency scenarios [91]. Figure 7 illustrates the principle of satellite-based thermal anomaly detection. Earth observation satellites equipped with thermal sensors capture surface temperature data and transmit the acquired information to ground-based servers for processing and analysis. Detected thermal anomalies are subsequently identified as potential active fire pixels. The lower panel presents an example of near-real-time global fire monitoring using NASA’s FIRMS platform, where active fire detections are highlighted in red.

To address these limitations, recent research has increasingly focused on machine learning and deep learning techniques applied to satellite imagery. Deep semantic segmentation methods using multi-sensor satellite data, including Sentinel and MODIS, have demonstrated improved accuracy in delineating wildfire-affected areas under both clear and cloudy conditions [92,93]. Machine-learning-based detection frameworks capable of operating day and night and interfacing with forest response systems via application programming interfaces (APIs) have further enhanced near-real-time wildfire monitoring [94]. Geostationary satellite platforms, such as GK2A, combined with random forest classifiers, have shown high detection accuracy and improved temporal resolution for wildfire monitoring in regional contexts [95].

Beyond detection, satellite-based systems have been integrated with semantic reasoning frameworks and linked geospatial data to improve data interoperability and decision support [96]. Specialized satellite missions, such as the BIRD satellite equipped with bi-spectral infrared sensors, enable high-resolution detection and quantitative characterization of wildfire parameters, including fire temperature, affected area, and radiative energy output [97]. Consequently, while satellite imaging alone may not fully satisfy real-time fire detection requirements, it remains a critical component of integrated wildfire monitoring systems when combined with ground-based sensors and aerial platforms.

### 3.6. Real-World Architectures

Modern forest fire detection systems are designed as hierarchical, multi-layer architectures that integrate sensing, communication, and processing components. Ground-based sensor nodes generate relatively low data volume and serve as the primary detection layer in operational deployments, continuously monitoring environmental parameters such as temperature, relative humidity, smoke concentration, and gas emissions. At this layer, nodes typically perform data acquisition and transmission without making definitive positive or negative fire decisions. These systems are optimized for low-power, long-term outdoor operation in remote forested environments.

In contrast to scalar environmental sensors, image-based sensors generate substantially larger data streams that require efficient processing and transmission strategies. Wherever an image sensor is deployed—whether on a fixed tower or an unmanned aerial vehicle (UAV)—a preliminary machine learning (ML) model may be implemented locally to reduce unnecessary transmission. For example, in [98], a TinyML model was implemented on a UAV platform to detect specific fire-related events and initiate image transmission only when a positive event was identified. Similarly, [99] demonstrated a multi-hop communication protocol in which information was forwarded sensor-to-sensor before reaching a cluster head for aggregation and further routing.

**Figure 8:**
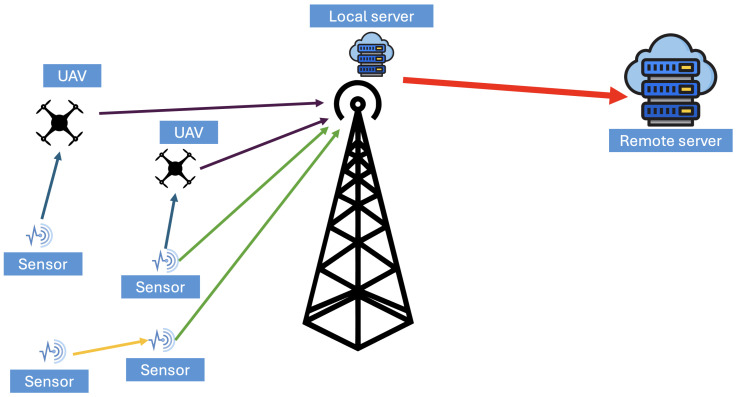
Hierarchical communication and data flow architecture of a forest fire detection system.

In practical deployments, the routing strategy depends on payload size, transmission distance, and the available communication technology. In short-range configurations, information may propagate in a node-to-node manner, where each device forwards measurements to its nearest neighbor. In geographically isolated areas, UAVs may periodically collect sensor streams from distributed ground nodes, functioning as mobile gateways. The collected information can then be transmitted from the UAV to a local server and subsequently forwarded to a cloud server for further analysis. In other deployment scenarios, ground nodes may transmit directly to a local server without intermediate relays. As illustrated in Figure 8, once information is collected at the sensing layer, it is transmitted through a communication infrastructure selected according to terrain conditions, infrastructure availability, bandwidth limitations, and energy constraints. In the figure, yellow arrows represent intra-communication between ground sensors, blue arrows denote short-range sensor-to-UAV links, green arrows indicate direct sensor-to-local-server transmissions, purple arrows represent UAV-to-local-server communication, and the red arrow illustrates backhaul transmission from the local server to the remote cloud server.

At the intermediate level, a local gateway or edge server aggregates information from multiple sensor nodes or aerial relays. Edge-level processing reduces latency and bandwidth consumption by performing preliminary anomaly detection and filtering before forwarding critical event summaries. The integration of edge computing within IoT architectures has been recognized as essential for time-sensitive environmental monitoring applications [100]. The local gateway is often equipped with multiple communication interfaces to ensure seamless exchange between sensor nodes and remote servers. At this stage, relevant measurements and summarized event information are transmitted to a cloud server through cellular backhaul, fiber-optic, or satellite communication links. Cloud platforms provide centralized storage, large-scale analytics, and integration with meteorological and geospatial databases. Advanced fire detection frameworks increasingly incorporate machine learning techniques applied to sensor and image data.

Performance metrics such as detection latency, accuracy, and deployment complexity vary across architectures because an FFD system integrates sensors, terrain conditions, communication networks, and ML or AI models deployed at the edge, local, or cloud levels. Ground-based scalar sensors can detect changes within seconds to minutes but cover limited areas. Satellite systems provide wide coverage but may experience delays from minutes to hours due to revisit intervals. Image-based systems often achieve high accuracy under controlled conditions, although performance can decline under poor visibility or harsh weather. Deployment complexity also differs, ranging from low-cost IoT nodes to infrastructure-intensive satellite and aerial platforms that require higher operational effort. Therefore, system performance must be evaluated in relation to its architectural design, and effective deployment requires balancing speed, coverage, accuracy, and practical constraints.

The final stage of the data flow involves decision support and alert dissemination. When detection confidence exceeds predefined thresholds, automated alerts are issued to forest management authorities and emergency response teams. Overall, operational forest fire detection systems rely on an integrated data pathway extending from distributed sensing nodes to gateways, edge processors, and cloud-based analytics platforms, where reliable communication and efficient multi-layer processing are essential for timely and accurate fire detection.

## 4. Data Transmission

Remote forest fire monitoring systems rely on a diverse set of sensors to acquire environmental, visual, and thermal data. Once collected, these data must be processed and transmitted in a manner that balances timeliness, reliability, energy consumption, and communication constraints. Depending on system architecture and application requirements, data analysis may be performed locally at the sensor node, partially at intermediate edge devices, or centrally at remote servers. In many deployments, outputs from multiple sensors are further combined to improve situational awareness and detection accuracy. Consequently, the choice of data transmission strategy plays a critical role in the overall effectiveness of remote environmental monitoring systems. Data processing in remote sensing systems can be broadly categorized into local (on-node), edge-level, and centralized processing paradigms. Local processing enables immediate analysis of raw sensor data at the node, reducing communication overhead and latency while conserving bandwidth and energy. Edge-based processing extends this concept by offloading computationally intensive tasks to nearby gateways or fog nodes, allowing partial aggregation, filtering, or fusion of sensor outputs before transmission to cloud servers. Centralized processing, while offering greater computational resources and long-term data storage, requires reliable communication links and incurs higher latency. Hybrid approaches that combine local feature extraction with edge or cloud-level decision-making have been shown to provide a favorable trade-off between efficiency and accuracy, particularly in multi-sensor wildfire monitoring applications [101,102].

Communication in remote forested environments presents significant challenges due to limited infrastructure, harsh terrain, and power constraints. Conventional cellular technologies such as LTE or GSM are often unavailable or unreliable in sparsely populated regions. Although Wi-Fi networks can be deployed locally, their limited range and high energy consumption restrict their applicability for large-scale environmental monitoring. These constraints necessitate the use of low-power, long-range communication technologies capable of supporting distributed sensor networks over wide geographic areas while operating on constrained energy budgets [103]. Low-Power Wide-Area Network (LPWAN) technologies, particularly LoRa and LoRaWAN, have emerged as promising solutions for long-range communication in remote sensing applications. LoRa employs chirp spread spectrum modulation to achieve transmission ranges of several kilometers while maintaining low power consumption. This makes it well-suited for periodic transmission of small sensor payloads such as temperature, humidity, gas concentration, and alert flags. However, the achievable data rate of LoRa is inherently low and subject to regulatory duty-cycle constraints, limiting its suitability for high-volume data transmission. Despite these limitations, LoRa-based systems have been widely adopted in environmental monitoring due to their robustness, scalability, and ease of deployment [104,105].

Transmitting high-volume data such as images or video over LoRa links remains a significant challenge. Even a single compressed full-resolution image may require tens of minutes to transmit when constrained by LoRa’s low data rate and duty-cycle regulations. Packet loss, channel variability, and energy limitations further complicate reliable image delivery. As a result, naïve transmission of raw or conventionally compressed multimedia data is impractical for LoRa-based systems. These constraints necessitate the development of transmission-aware data reduction strategies that minimize payload size while preserving critical information for fire detection and assessment [106]. Conventional image and video compression standards, such as JPEG or H.264, are designed for high-bandwidth channels and do not perform optimally under the severe constraints imposed by LoRa networks. To address this gap, LoRa-oriented compression techniques have been proposed that emphasize progressive transmission, region-of-interest prioritization, and lightweight encoding. Approaches such as bit-plane decomposition, block-based differential encoding, run-length encoding, and entropy coding enable gradual reconstruction of images while allowing early termination once sufficient information has been received. Progressive and hierarchical transmission schemes are particularly attractive, as they permit coarse scene understanding from partial data and reduce retransmission overhead in lossy channels. These specialized compression strategies enable practical image-based monitoring over LPWAN links and are increasingly explored in wildfire detection and remote sensing research [107].

**Figure 9:**
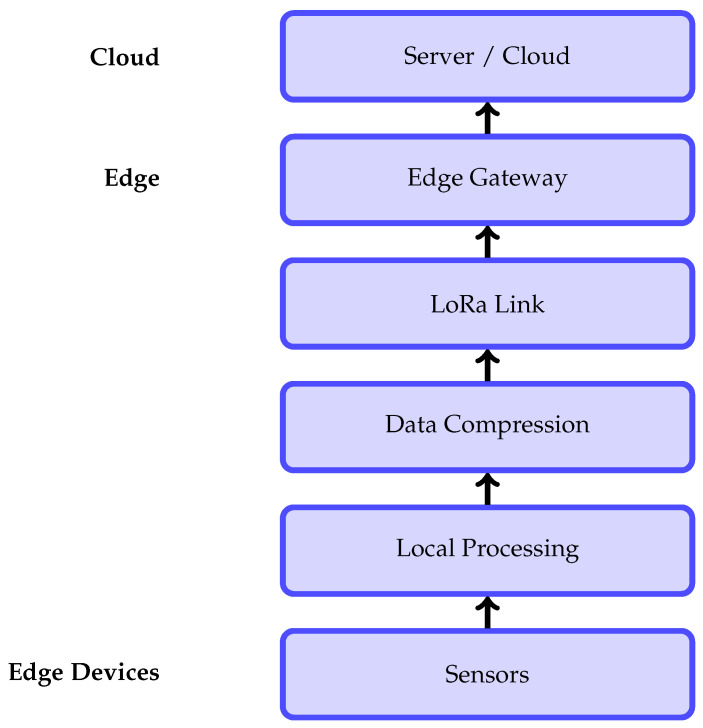
Data transmission pipeline from sensor nodes to cloud infrastructure, illustrating local processing, compression, LoRa-based communication, and edge-to-cloud data transfer.

Communication technology selection is a critical design consideration for remote environmental monitoring systems, as it directly affects coverage, energy consumption, latency, and achievable data volume. As summarized in Table 3, conventional high-bandwidth solutions such as Wi-Fi and cellular networks offer low latency and high data rates but are limited by short range, infrastructure dependency, and high power consumption. In contrast, LPWAN technologies such as LoRa provide long-range connectivity with minimal energy requirements, making them well-suited for remote and infrastructure-sparse environments. However, the very low data rate and relatively higher latency of LoRa significantly constrain the transmission of high-volume data, such as images or video, thereby motivating the need for transmission-aware data reduction and compression strategies. As illustrated in Figure 9, sensor data are locally processed and compressed prior to LoRa-based transmission to an edge gateway, followed by cloud-level analysis.

## 5. Data Post-Processing

**Figure 10:**
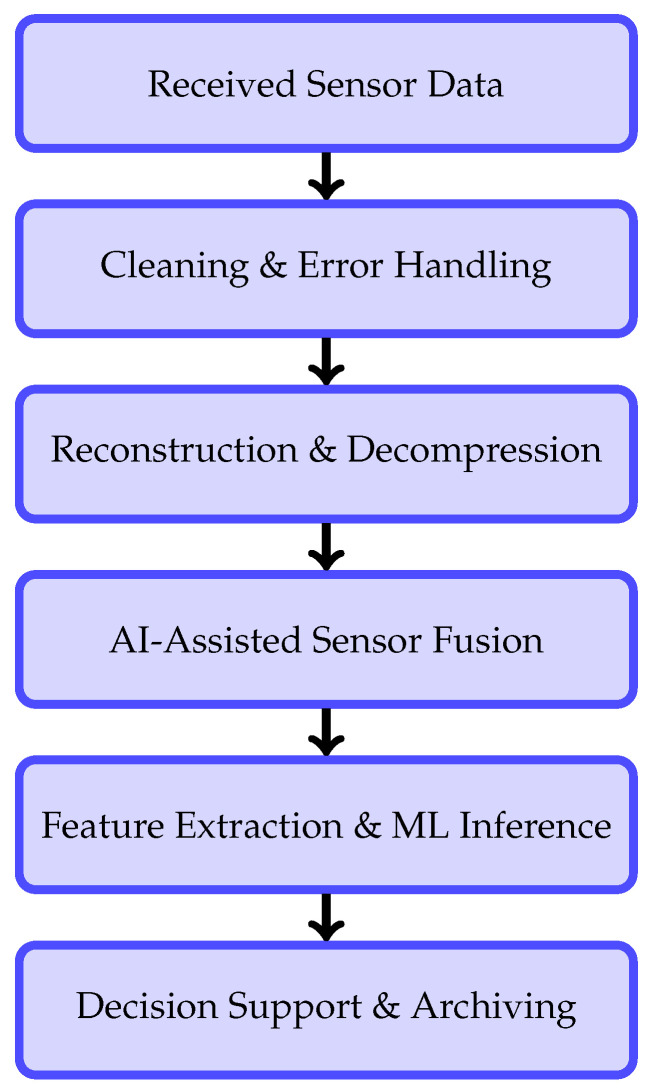
Post-processing pipeline for remote wildfire monitoring systems incorporating AI and machine learning.

After data transmission, post-processing plays a critical role in transforming raw and compressed sensor data into reliable and actionable information. In remote wildfire monitoring systems, post-processing must account for transmission constraints, sensor noise, packet loss, and partial data recovery, particularly when LPWANs are employed. Moreover, the integration of artificial intelligence (AI) and machine learning (ML) techniques has become increasingly important to enhance robustness, accuracy, and adaptability in fire detection and situational assessment. An overview of the main post-processing functions involved in this stage is summarized in Table 4, while the overall processing workflow is illustrated in Figure 10.

### 5.1. Data Cleaning, Noise Suppression, and Error Handling

Environmental sensors deployed in outdoor conditions are subject to noise caused by atmospheric variations, sensor drift, and hardware limitations. Additionally, low-bandwidth communication links may introduce packet loss, quantization errors, or out-of-order data delivery. To mitigate these effects, post-processing pipelines typically apply filtering and smoothing techniques, such as moving averages, median filters, and statistical outlier rejection, to stabilize scalar sensor measurements. Error detection mechanisms and basic redundancy checks are also employed to identify corrupted or missing packets before further analysis. These operations constitute the first stage of the pipeline shown in Figure 10.

### 5.2. Data Reconstruction and Decompression

Data transmitted over LoRa links are often fragmented and heavily compressed to meet strict payload size constraints. Post-processing therefore includes packet reassembly and decompression to reconstruct sensor streams or image data. Progressive reconstruction strategies allow partial data to be utilized even when full payloads are unavailable, enabling early situational awareness under unreliable network conditions [106]. This reconstruction stage bridges the gap between raw received data and higher-level analysis, as indicated in Figure 10.

### 5.3. AI-Assisted Multi-Sensor Data Fusion

Wildfire detection systems increasingly rely on heterogeneous sensing modalities, including temperature, humidity, gas concentration, thermal imaging, and optical sensors. Post-processing aligns these data streams temporally and spatially before fusion. AI-assisted fusion techniques combine complementary sensor information to reduce false alarms and improve detection confidence, particularly under ambiguous conditions such as fog, dust, or variable lighting [71]. As summarized in Table 4, data fusion serves as a key intermediate step that enhances the reliability of subsequent feature extraction and decision-making processes.

### 5.4. Feature Extraction and Machine Learning-Based Event Detection

Once data are reconstructed and fused, relevant features are extracted to characterize fire-related events. These features may include temporal trends, spatial gradients, anomaly indicators, and texture or intensity descriptors in the case of image data. Machine learning models are then applied to classify events, detect anomalies, or predict fire risk levels. ML-based approaches are particularly effective in handling noisy and incomplete data, offering improved adaptability compared to fixed threshold-based methods.

### 5.5. Decision Support, Alert Generation, and Data Archiving

The outputs of post-processing and ML inference are integrated into decision-support mechanisms that generate alerts and notifications. Confidence scoring and prioritization strategies are applied to reduce false positives and ensure timely response. Processed data and detection outcomes are archived for long-term analysis, system evaluation, and model retraining, supporting continuous improvement of wildfire monitoring performance.

## 6. Challenges and Limitations

Although FFD systems have advanced considerably in recent years, several challenges and limitations continue to hinder their reliable and large-scale deployment. These challenges primarily arise from harsh environmental conditions, stringent energy requirements, sensor deployment constraints, and communication limitations inherent to remote and forested regions.

### 6.1. Environmental Challenges

Environmental variability significantly affects the performance and reliability of FFD systems. Adverse weather conditions, including fog, rain, snow, and strong winds, degrade the effectiveness of optical and thermal sensors by obscuring visual cues and attenuating thermal signatures, thereby increasing the likelihood of missed detections and false alarms [108]. In addition, complex terrain features and dense vegetation often obstruct line-of-sight sensing, limiting the effectiveness of vision-based systems and reducing the accuracy of satellite-based monitoring in forested environments [73]. Seasonal variations further complicate detection, as changes in fuel moisture content, vegetation density, and ambient temperature directly influence fire behavior and sensor response, introducing uncertainty in detection thresholds and system calibration [109].

### 6.2. Sensor Deployment and Coverage Constraints

Beyond sensing and energy considerations, the effective deployment of sensors across large forested areas presents a significant challenge [110]. Sensor deployment strategies must balance inter-node distance, spatial coverage, redundancy, and terrain constraints to ensure reliable detection while minimizing cost and energy consumption [111]. Inadequate sensor placement may lead to coverage gaps, delayed detection, or excessive redundancy, while heterogeneous terrain, dense vegetation, and limited accessibility in remote regions further complicate optimal sensor density and placement, directly impacting network connectivity, data reliability, and system robustness [61].

### 6.3. Limitations of Data Transmission

Several limitations inherent to existing technologies further restrict the effectiveness of FFD systems. Vision-based sensing platforms generate large volumes of image and video data, which are challenging to transmit over low-power, long-range wireless networks commonly used in remote monitoring scenarios [112]. Most current systems employ conventional image compression techniques optimized for high-bandwidth communication links, rendering them unsuitable for ultra-low-bandwidth and energy-constrained technologies such as LoRa [113]. Additionally, long-range IoT technologies impose strict constraints on payload size, duty cycle, and airtime, severely limiting real-time image transmission and continuous monitoring capabilities [112]. Meanwhile, satellite-based monitoring provides broad spatial coverage; however, the trade-offs between spatial resolution, revisit frequency, and data latency restrict its suitability for early-stage fire detection and rapid response [109].

### 6.4. Energy Constraints in Ground Sensor Networks

One of the most critical technical challenges in remote FFD deployments is the lack of sustained energy availability. Many existing systems rely on battery-powered or grid-dependent sensor nodes, which severely limit long-term operation in remote and inaccessible forest regions [114]. For persistent monitoring, energy-autonomous sensor systems capable of harvesting ambient energy—such as solar, thermal, or vibrational energy are essential. However, despite extensive research efforts, fully energy-autonomous wireless sensor networks remain largely absent in real-world FFD deployments due to intermittent energy sources, limited storage capacity, and increased system complexity [115]. Furthermore, automated fire detection algorithms are highly sensitive to illumination changes, environmental noise, and long-term sensor drift, which can result in elevated false-positive and false-negative rates under real operating conditions [73].

### 6.5. Energy Sustainability Challenges in UAV-Based Monitoring

Sustained UAV-based wildfire surveillance requires reliable and continuous access to energy, making recharging and energy sustainability a critical operational challenge. Standalone UAV missions typically require multiple recharging or refueling stations to maintain persistent coverage over large forested areas, which increases deployment complexity, infrastructure costs, and logistical overhead [116]. In parallel, ground-based sensor networks supporting wildfire detection demand uninterrupted power for sensor nodes and local communication hubs to enable multi-hop routing and reliable uplink transmission to central servers [117].

Because both UAV platforms and sensor nodes are commonly deployed in remote and inaccessible environments, routine battery replacement or manual recharging is often impractical. As a result, ensuring a sufficient and sustainable energy supply for sensing, onboard processing, and scheduled data transmission remains one of the most significant barriers to long-term autonomous operation [118]. Energy harvesting technologies have therefore attracted increasing attention as a means to extend system lifetime by exploiting ambient energy sources such as solar radiation, thermal gradients, wind-induced vibration, and radio-frequency energy [119,120].

Despite their availability in natural environments, individual energy harvesting sources often provide intermittent and limited power, which is insufficient to support continuous UAV operation or dense wireless sensor networks under realistic workloads. Achieving practical energy autonomy typically requires modifications to traditional energy conversion architectures, efficient power management strategies, and the hybrid integration of multiple energy harvesting mechanisms combined with energy storage elements [121]. Although notable progress has been made in laboratory-scale prototypes and short-term field deployments, fully energy-sustainable UAV-assisted sensing systems remain largely absent in operational wildfire monitoring scenarios. Consequently, the design of energy-aware architectures and energy-sustainable aerial–ground sensing platforms continues to be a primary focus of ongoing research.

## 7. Conclusions

This work reviews the development of forest fire detection systems. It demonstrates how monitoring has evolved from manual observation and fixed surveillance to distributed sensing architectures that integrate ground sensors, communication networks, and automated analysis. Although recent technological advances have improved early fire detection, reliable operation in remote forest environments remains limited by communication bandwidth, energy availability, and deployment constraints. Long-range, low-power networks such as LoRa provide connectivity in areas without conventional infrastructure. However, their low data rates make the transmission of visual information challenging. This limitation necessitates image compression and reconstruction techniques tailored to LoRa-class links rather than conventional high-bandwidth systems. At the same time, long-term wildfire monitoring cannot depend on frequent battery replacement or grid power, making energy-autonomous sensor nodes based on energy harvesting and adaptive duty cycling essential for practical deployment. Addressing these constraints jointly—data efficiency, communication limits, and energy autonomy—is essential for developing scalable and reliable forest fire detection systems. Such systems must operate sustainably in remote and high-risk environments.

## Figures and Tables

**Table 1:** Representative characteristics of commonly used in situ sensors for forest fire detection (FFD) systems.

Sensor Type	Measured Parameter	Typical Range	Primary Role in FFD
Temperature/Humidity (DHT22)	Air temperature, RH	−40–80 °C; 0–100% RH	Fire-risk assessment and ambient condition monitoring
Infrared Flame Sensor	IR radiation (flame emission)	Up to ∼1–5 m (line-of-sight)	Local flame presence confirmation
Environmental Sensor (BME280)	Temperature, RH, pressure	−40–85 °C; 0–100% RH; 300–1100 hPa	Weather trend analysis and ignition risk modeling
Wind Speed Sensor	Wind velocity	0–30 m/s (typical)	Fire spread estimation and direction modeling
CO_2_ Sensor (NDIR)	Carbon dioxide concentration	400–5000+ ppm	Smoldering fire and combustion gas detection
Smoke/Gas Sensor (MQ-2)	Combustible gases, smoke	Qualitative/relative	Early-stage smoke and gas indication

**Table 2:** Comparison of imaging sensor modalities for forest fire detection (FFD).

Features	RGB Camera	Thermal Camera	Multispectral Camera
Primary observable	Smoke, flame, visual changes	Heat anomalies, hotspots	Vegetation stress, moisture, spectral changes
Fire stage sensitivity	Early smoke and visible flame	Active fire and hotspots	Pre-fire risk and early stress
Nighttime operation	No	Yes	Limited (band-dependent)
Sensitivity to weather	High (fog, rain, lighting)	Moderate (smoke penetration)	Moderate to high (cloud cover)
False alarm sources	Clouds, dust, shadows	Hot objects, sun-heated surfaces	Seasonal and phenological variation
Spatial coverage	High	Moderate	Very high
Processing complexity	Low–moderate	Moderate	High
Typical deployment	Towers, UAVs	UAVs, ground stations	UAVs, satellites
Role in FFD systems	Early visual detection	Fire confirmation and tracking	Risk assessment and prevention

**Table 3:** Comparison of communication technologies for remote environmental monitoring.

Technology	Range	Data Rate	Latency	Power	Typical Use Case
Wi-Fi	∼100 m	11–450 Mbps	1–100 ms	100–500 mW	Local sensing and short-range data transfer
Cellular (LTE/5G)	∼35 km	10–100 Mbps	10–150 ms	500 mW–3 W	Urban and semi-urban environmental monitoring
LoRa/LoRaWAN	5–15 km	0.3–50 kbps	1–10 s	10–100 mW	Remote, low-power sensor networks
Satellite	Global	∼1 Mbps	0.5–2 s	5–50 W	Emergency communication and sparse coverage areas

**Table 4:** Key post-processing functions in remote wildfire monitoring systems.

Function	Description
Data Cleaning	Noise suppression, outlier removal, and handling missing or corrupted packets
Reconstruction	Packet reassembly and decompression of sensor and image data
Data Fusion	Integration of heterogeneous sensor streams for improved reliability
Feature Extraction	Derivation of fire-relevant indicators from reconstructed data
Decision Support	Alert generation, prioritization, and system feedback

## Data Availability

Not applicable.
